# Large scale controlled Fab exchange GMP process to prepare bispecific antibodies

**DOI:** 10.3389/fbioe.2023.1298890

**Published:** 2024-01-12

**Authors:** Xia Yao, Mingquan Xie, Yinyin Ben, Yixiang Zhu, Gaoqiang Yang, Simon Chi Wai Kwong, Zhengliang Zhang, Mark L. Chiu

**Affiliations:** ^1^ Tavotek Biotherapeutics, Suzhou, China; ^2^ Bioworkshops (Suzhou) Limited, Suzhou, China; ^3^ Tavotek Biotherapeutics, Lower Gwynedd, PA, United States

**Keywords:** bispecific antibodies, manufacturing production, controlled Fab-arm exchange, antibody expression, purification

## Abstract

**Objective:** Bispecific antibodies (BsAbs) have demonstrated significant therapeutic impacts for the treatment of a broad spectrum of diseases that include oncology, auto-immune, and infectious diseases. However, the large-scale production of clinical batches of bispecific antibodies still has many challenges that include having low yield, poor stability, and laborious downstream purification processes. To address such challenges, we describe the optimization of the controlled Fab arm exchange (cFAE) process for the efficient generation of BsAbs.

**Methods:** The process optimization of a large-scale good manufacturing practice (GMP) cFAE strategy to prepare BsAbs was based on screening the parameters of temperature, reduction, oxidation, and buffer exchange. We include critical quality standards for the reducing agent cysteamine hydrochloride.

**Results:** This large-scale production protocol enabled the generation of bispecific antibodies with >90% exchange yield and at >95% purity. The subsequent downstream processing could use typical mAb procedures. Furthermore, we demonstrated that the bispecific generation protocol can be scaled up to ∼60 L reaction scale using parental monoclonal antibodies that were expressed in a 200 L bioreactor.

**Conclusion:** We presented a robust development strategy for the cFAE process that can be used for a larger scale GMP BsAb production.

## 1 Introduction

In the last 30 years, antibody-based drugs represent an important class that is widely used in a broad range of clinical treatment for autoimmune, metabolic, and oncology diseases. In contrast to monoclonal antibodies that can bind only a single target, bispecific antibodies can recognize two different antigens at the same time, thereby increasing therapeutic utility to combat different diseases (Wang et al., 2019). Since the first bispecific antibodies (bsAb), catumaxomab and blinatumomab were approved, more than 200 bsAbs are under preclinical or clinical investigation. Many bsAbs are designed to redirect and activate CD3-expressing (Cluster of Differentiation 3) cytotoxic T cells against cancer cells, while others target immune checkpoints, oncogenic signaling pathways, and cytokines (Mullard, 2017; Betts and van der Graaf, 2020). Given that bsAbs are still of increasing interest for therapeutic applications, more efficient methods to generate recombinant bsAbs with defined biochemical and pharmacological properties are necessary (Tiller and Tessier, 2015).

Unlike the parental mAbs, production challenges from a practical and cost-effective manner, have hampered bsAbs from being efficiently produced at the bench or at large scale. Heavy chain and light chain mispairings in products have been reported as one of major sources of impurities during bsAbs production, which can hinder downstream processing (Klein et al., 2012). To increase the purity and yield of recombinant bsAbs, several strategies were developed to produce bsAbs, including: knobs-into-holes technology (Ridgway et al., 1996), common light chain (De Nardis et al., 2017), CrossMab technology (Klein et al., 2019), and quadroma technology (Zhang et al., 2017). Although these advanced strategies can remove the mispaired byproducts for the subsequent downstream processing, multiple purification steps and complicated design are needed, which are often laborious or problematic.

The controlled Fab-arm exchange (cFAE) is an efficient process to generate bispecific IgG1 by taking two parental mAbs to recombine and form stable bsAbs, albeit at a small scale (Van Der Neut Kolfschoten et al., 2007; Gramer et al., 2013; Labrijn et al., 2013; Labrijn et al., 2014). The introduction of two matched point-mutations, F405L and K409R, into the CH3 domains of two parental mAbs, respectively. Upon mild reducing conditions, these point-mutations drive the formation of the BsAb heterodimer and locked into the final conformation upon the re-oxidation condition. Compared to the other BsAb preparation methods, cFAE can generate bsAbs in a fast and applicable process. There are two FDA approved bsAbs that are produced by the cFAE method: Amivantamab (EGFR/cMET) (Neijssen et al., 2021) and Teclistamab CD3/B Cell Maturation Antigen (BCMA) (Pillarisetti et al., 2020). While cFAE has become one of the more popular methods to generate bsAbs, there is limited information about the scale-up of cFAE process at the good manufacturing practice (GMP) manufacturing scale.

In this report, we evaluated the cFAE process parameters of reaction time, pH, temperature, residual reductant removal procedure, and oxidation time. We also outlined the optimization of cFAE process by varying pH, temperature, time for reduction, oxidation, and diafiltration. We describe a process that was used to generate bsAbs with high purity and yield from parental mAbs produced in the 200 L manufacturing scale. Furthermore, we demonstrated that bsAbs can be generated with purity more than 95% and parental mAb exchange yield of 90%.

## 2 Materials and methods

### 2.1 Cells

The host Chinese Hamster Overlay (CHO) cell line was cultured in medium of BalanCD CHO Growth A (Irvine scientific, Cat. No. 94120).

### 2.2 Cloning and production of parental mAbs

Relevant expression vectors containing the heavy and light chains of parental antibodies were transfected into CHO host cells to prepare recombinant CHO cell lines, respectively. Each production process included independent cell line seed training and fed-batch cell culture in a 200L GE Xcellerex XDR-200 single-use bioreactor (as workflow described in Figure 7). The chemical-defined basal medium of BalanCD CHO Growth A and feed mediums of Cell boost 7a, 7b (Cytiva, Cat. No. SH31026 and SH31027) were used as production media, respectively. The harvested cell fluid was then clarified and filtered, followed by Protein A affinity chromatography (Cytiva, Cat. No. 175474) and intermediate depth filtration. The target bsAb was then assembled *in vitro* from parental antibodies via 2-MEA (2-mercaptoethylamine) cFAE reaction. The target bsAb was subjected to a platform antibody manufacturing process, including ultrafiltration/diafiltration (UF/DF), oxidation, low pH virus inactivation, chromatograph polishing, and nano virus filtration. UF/DF was used to concentrate the protein before the final formulation and excipients were added to formulate the final drug substance.

### 2.3 Process optimization for cFAE reaction pH, temperature, time

Protein samples from 15 L/50 L bench-scale bioreactors were used for cFAE reaction process optimization. The cFAE reaction was mediated by 75 mM 2-MEA (Sigma, Cat. No. 30078) in buffer pH 5.5 or 7.5 to an equal amount of parental antibody A and B. The samples were divided into two portions, one placed in a water bath at 18°C and the other placed in a water bath at 26°C. Sampling was performed at reaction timepoints of 5, 8, 12, and 24 h, respectively, followed by a diafiltration step to remove the reducing agent and then placed at room temperature for oxidation. Retained samples were then analyzed by CE-NR, SEC-HPLC (High Performance Liquid Chromatography) and/or CEX-HPLC testing for target product quality.

### 2.4 Effect of oxidation time on the cFAE reaction

Parental antibody A and B were generated from 15 L to 50 L bench-scale bioreactor, respectively, with harvest titers of 1.0 g/L. Proteins were purified by affinity chromatography and intermediate depth filtration. The final reaction solution contained 75 mM 2-MEA and 3.2 g/L of each parental antibody A and B in 620 mL buffer, pH 7.5. The cFAE reaction took 8 h in a closed container at room temperature when mixing well. The sample was concentrated to target concentration of 15.0 g/L by ultrafiltration membrane pack followed the reducing agent was removed by diafiltration exchange with 10 times volume of the concentrated samples. The samples were sterile filtered into 500 mL bottles and oxidized at room temperature with air on the sample surface and stirred continuously at 30 rpm, and the samples were also sampled for product quality analysis at 15, 20, 24, 37, and 48 h of each oxidation timepoint.

### 2.5 2-MEA removal study

Parental antibody A and B were generated from 50 L bench-scale and 200 L bioreactor production batches, respectively. Proteins were purified by affinity chromatography and intermediate depth filtration. The cFAE reaction was mediated by 2-MEA (Sigma, Cat. No. 30078) in buffer, pH 7.5 with an equal amount of parental antibody A and B. The final reaction solution contained 75 mM 2-MEA and an equal amount of parental antibody A and B in 331.0 mL of buffer, pH 7.5. The reaction took 10 h in a closed container at room temperature. The reducing agent was removed by diafiltration using a 0.11 m^2^ Biomax Polyether sulfone (PES) ultrafiltration membrane (30 kDa molecular weight cut-off, Millipore), with an inlet flow rate of 271 L per square meter per hour (LMH) and a trans-membrane pressure drop (TMP) of 0.7 bar. Samples were retained at 5, 8, 10, 12, 14, 16, 18, and 20 times of the exchange buffer volume.

### 2.6 Scale-up of cFAE

The cFAE reaction was mediated by adding 5.78 L of a 750 mM stock solution of 2-MEA in buffer (pH 7.3–7.7) to an equal amount of parental antibody A and B. The final reaction solution contained 75 mM 2-MEA and 3.2 g/L of parental antibody A and B in 57.8 L of buffer (pH 7.3–7.7). After 9 h of reaction in a closed container at ambient temperature (18°C–26°C), the samples were concentrated to a protein concentration of 15 g/L by ultrafiltration membrane, and the reducing agent was then removed by diafiltration exchange with 10 times the diafiltration volume.

### 2.7 Titer by protein A HPLC

The titer of cell culture broth was evaluated on a Waters e2695 Affinity chromatography HPLC instrument or equivalent using MAbPac Protein A column (4 × 35 mm). Mobile phase A was phosphate buffer at neutral pH (50 mmol/L Phosphate buffer, 150 mmol/L NaCl, 5% Acetonitrile, pH 7.5), while mobile phase B was phosphate buffer at acidic pH (50 mmol/L Phosphate buffer, 150 mmol/L NaCl, 5% Acetonitrile, pH 2.5). The HPLC system and the chromatographic column were equilibrated with 100% mobile phase A, at a flow rate of 2.0 mL/min until the absorbance baseline was stable. Then, running the system with 100% B for elution for 2 min was performed. Reference and sample were directly injected, and the injection volume was 20 μL. Absorbance figures were analyzed at 280 nm using the Empower 3 system or other integration software. External standard method was used to quantify the test samples. A series of reference standard solutions with known concentrations were injected into the HPLC system. The peak areas were measured and plotted against the corresponding concentrations to create a calibration curve, enabling the determination of test sample concentrations through comparison with the curve.

### 2.8 Cation exchange chromatography

The levels of protein charge variances were assessed on a Waters e2695 Cation Exchange Chromatography (CEX) HPLC instrument or equivalent using YMC BioPro SP-F column (5 μm, 100 × 4.6 mm). Mobile phase A was composed of 20 mM Phosphate buffer, while mobile phase B was composed of 0.3 mol/L sodium chloride in Phosphate buffer. The percentage of mobile phase B increased from 20.0% to 100.0% and 100.0% to 20.0% in 22.0 and 24.0 min, respectively. Absorbance figures were analyzed at 280 nm using the Empower 3 system.

### 2.9 Residual CHO host cell protein

Levels of residual CHO host cell protein (HCP) were measured with an enzyme linked immunosorbent assay (Kit from Cygnus, F550-1). Samples were reacted simultaneously with a horseradish peroxidase (HRP) enzyme labeled anti-CHO antibody in microtiter strips coated with an affinity purified capture anti-CHO antibody. The immunological reactions form a sandwich complex of solid phase antibody-HCP-enzyme labeled antibody. Then the microtiter strips were washed to remove the unbound reactants and the substrate TMB (3,3,5,5-Tetramethyl Benzidine) was reacted. Read plate with absorbance setting at OD_450/600 nm._ The amount of hydrolyzed substrate was measured to be directly proportional to the concentration of CHO HCPs in the sample.

### 2.10 Residual CHO host cell DNA

Residual host cell DNA was measured using a commercial quantitative real-time polymerase chain reaction (qPCR) kit (SHENTEK, SK030203D100). Total DNA was extracted from the test sample and amplified using PCR. During qPCR reaction, successive cycles of template denaturation, primer annealing, and product extension amplify the target sequence. The reporter dye was released from the probe and generate fluorescent signal during amplification. When the fluorescence intensity released during the reaction reaches the preset threshold value, the number of PCR cycles have a linear relationship with the logarithmic value of initial DNA template from the system and the fluorescent signal was then detected. The amount of target DNA was determined by comparing the fluorescent intensity of the sample to a standard curve.

### 2.11 Residual protein A

Residual Protein A was measured using a commercial Enzyme Linked Immunosorbent Assay kit (Repligen, 9222-1). Samples and serial diluted standards were added to 96-well plates, followed by addition of Rabbit anti-Protein A and Streptavidin-HRP conjugate. After a wash step to remove unbound reactants, TMB substrate was added to the sandwiched complex. The reaction was terminated by stop solution. Read plate with absorbance setting at OD_450/600 nm_. The amount of residual Protein A in the sample was calculated based on the standard curve.

### 2.12 Endotoxin

Bacterial endotoxins were measured using the gel-clot assay in compliance with USP <85>.

### 2.13 Bioburden

The bioburden was measured using the membrane filtration method in compliance with USP <61>.

### 2.14 Ultraviolet-visible spectroscopy

Protein content was measured by Ultraviolet-visible (UV) spectroscopy absorbance at 280 nm. Ultra-purity water was used as sample dilution buffer and blank. The protein content was calculated following the formula below:

$$\text{Protein content }\left(\frac{mg}{mL}\right)=\text{Absorbance mean value}\times \frac{\;\text{Total}\;\text{dilution}\;\text{factor}}{\text{Theoretical}\;\text{extinction}\;\text{coefficient}}$$

The theoretical extinction coefficient of the parental mAb A, B and target bsAb were all 1.47 (mg/mL)^−1^·cm^−1^.

### 2.15 Size exclusion chromatography

The sample purity by size exclusion chromatography (SEC) was monitored on a Waters e2695 HPLC instrument or equivalent, using a TOSOH TSK gel G3000SWxl SEC column (5 μm, 7.8 mm I.D. ×30 cm). The mobile phase was 200 mM KH_2_PO_4_, 150 mM KCl at pH 6.8, with a flow rate of 0.5 mL/min. The test sample amount was adjusted to 100 μg before injection, and absorbance figures were analyzed at 280 nm using the Empower 3 system or other integration software.

### 2.16 SDS capillary electrophoresis (reduced and non-reduced)

The sample purity by capillary electrophoresis sodium dodecyl sulfate (CE-SDS) was evaluated by PA800 Plus equipment from SCIEX. Sample preparation was executed with the Protrome Lab SDS-Gel molecular weight analysis chemistries kit according to the vendor’s manual. Non-reduced samples were denatured with N-ethylmaleimide (NEM) at 70°C for 5 min, while reduced samples were denatured with β-Mercaptoethanol (β-ME) at 70°C for 10 min. After cooling, the samples were electrokinetically introduced into the capillary and separated by constant voltage. Components of different molecule sizes in the protein samples were detected as they pass through the capillary with a Photo-diode array detector at 220 nm.

### 2.17 2-MEA content testing by FLD-UPLC

2-MEA residue was monitored on Thermo Vanquish UPLC instrument using Waters BioResolve-RP-mAb Polyphenyl column (2.7 μm, 2.1 × 100 mm). Mobile phase A was composed of 99.9% water and 0.1%TFA, while mobile phase B was composed of 90%ACN, 9.9%water and 0.1%TFA. The percentage of mobile phase B increased from 1.0% to 5.0% and 5.0%–99.0% in 3.5 and 6.0 min, respectively. The signal of 2-MEA was detected using an expiation spectrum at 266 nm and an emission spectrum at 473 nm by Fluorescence Detector. This quantification was conducted using the external standard method.

### 2.18 2-MEA identification by infrared spectroscopy (IR)

Infrared spectroscopy was used to identify the 2-MEA. 1.5 mg of the tested sample was ground in an agate mortar with 300 mg of dry potassium bromide (KBr), mixed thoroughly and then placed in a 13 mm diameter cylindrical sample cup in pressing mold. The mixture was held for 2 min with a pressure of 12.5∼15.6 MPa. The prepared article was then evaluated using a Thermo IS 10 Fourier-Transform Infrared spectrometer (FT-IR).

### 2.19 Cyclic voltammetry testing of 2-MEA

The electrochemical workstation Metrohm PGSTAT302N was used to perform cyclic voltammetry. 0.1 g of the test article was dissolved in 100 mL of a 1 mol/L of KCl solution as the electrolyte, and a Glassy carbon electrode was used as working electrode, a 1 cm^2^ platinum sheet as the counter electrode, and a saturated calomel electrode as reference electrode. The electrochemical workstation Metrohm PGSTAT302N was used to complete a cycle at a scanning rate of 10 mV/s between the potential range of 0.6 V and −0.8 V. The tested article was oxidized by applying a positive potential to the working electrode surface, and the reduction reaction of tested article occurred by applying a negative potential to the working electrode surface.

### 2.20 Cyclic voltammetry testing of 2-MEA

The electrochemical workstation Metrohm PGSTAT302N was used to perform cyclic voltammetry. 0.1 g of the test article was dissolved in 100 mL of a 1 mol/L of KCl solution as the electrolyte, and a Glassy carbon electrode was used as working electrode, a 1 cm^2^ platinum sheet as the counter electrode, and a saturated calomel electrode as reference electrode. The electrochemical workstation Metrohm PGSTAT302N was used to complete a cycle at a scanning rate of 10 mV/s between the potential range of 0.6 V and −0.8 V. The tested article was oxidized by applying a positive potential to the working electrode surface, and the reduction reaction of tested article occurred by applying a negative potential to the working electrode surface.

### 2.21 Detection of bsAb by liquid chromatography mass spectrometry (LC-MS)

The intact molecular weight of antibodies was assessed using Thermo Vanquish UPLC combined with Q Exactive HF-X MS and a Waters RP column (BioResolve-RP-mAb Polyphenyl, 450 Å, 2.1 × 100 mm, 2.7 µm). The mobile phase A (0.12% FA in water) and the mobile phase B (0.12% FA in ACN) were used in this method. The system and the chromatographic column were equilibrated with 80% mobile phase A, at a flow rate of 0.2 mL/min until the absorbance baseline was stable. Then, a gradient of 20% B to 80% B elution program for 7 min was performed. Samples were deglycosylated using peptide-N-glycosidase F (New England Biolabs, Cat. No. P0704L) enzyme and incubated at 37°C for 4 h with a final concentration of approximately 0.4 mg/mL. The injection volume was 5 μL. The detection wavelength was 214 nm. Column temperature was 60°C. The m/z scanning range was 2,000–4,000.

### 2.22 Enzyme linked immunosorbent assay (ELISA)

Binding activity of bsAbs was measured by ELISA binding. The test samples and reference standard of bsAbs were serial diluted. Antigen was diluted to 0.5 μg/mL and coated on 96-well ELISA plates and incubated overnight, then the plate was washed and blocked with commercial block buffer (Sangon). The diluted test samples were added to the wells and incubated at room temperature for 2 h. After the plates were washed, the Peroxidase AffiniPure F (ab’)₂ Fragment Goat Anti-Human IgG, Fcγ fragment specific antibody (Jackson Immuno Research) was added to the plates and incubated at room temperature for 1 h. After the plates were washed, the TMB solution was added to the plates. The reaction was terminated using a stop solution. Read plate with absorbance setting at OD_450/600 nm_. A four-parameter logistic curve fit was used to calculate the EC50 values of the samples. The binding activity of samples was reported in percentage relative to the activity of the reference standard.

## 3 Results

### 3.1 Parental antibody manufacturing

The parental antibodies IgG1 A and B were generated for further cFAE reaction using the respective recombinant Chinese Hamster Overlay (rCHO) cell lines. Separate rCHO cell lines encoded with parental A with a F405L mutation and B antibody with a K409R mutation were constructed, respectively. Each of the two parental antibodies were produced in separate 200 L single use bioreactors. The cell culture growth profiles showed good culture conditions for the cells growing in each production process, with a maximum cell density of 33∼41 million viable cells per mL (Table 1). Both manufacturing processes ended on day 11, and each of the parental antibodies were recovered from the bioreactor by a 2-step filtration to provide the clarified cell culture fluid. The clarified fluid was then put through a Protein A affinity chromatography step to isolate the respective antibodies. Further process impurities were removed after an intermediate depth filtration step.

**TABLE 1:** Summary of parental antibody production and harvest in 200 L bioreactor.

Parental antibody	Maximum VCD	Duration	End viability (%)	Final titer	Total protein produced in bioreactor^a^ ^(g)^	Harvest recovery^b^ ^(%)^
A	41.3 × 10^6^ cells/mL	11 days	93.1	1.88 g/L	340	89.8
B	33.9 × 10^6^ cells/mL	11 days	89.8	1.36 g/L	256	88.7

Note: VCD, indicated viable cell density; VCD, and viability were analyzed by Vi-cell cell viability analyzer (Beckman); Duration indicated the cell culture production days; Final titer was tested by Protein A-HPLC, method.

^a^ Total protein produced of parental antibody A (340 g) was calculated as equal to a final titer of 1.88 g/L and a harvest volume of 180.6 kg, while the total protein amount of parental antibody B (256 g) was equal to a final titer of 1.36 g/L and a harvest volume of 188.6 kg.

^b^ Harvest recovery was calculated as the product ratio of total protein collected after clarification to the total protein produced in bioreactor (as above 1).

In the Parental antibody A case, harvest recovery of parental antibody A = Total protein collected after clarification/Total protein produced in bioreactor = Product of harvest volume and harvest titer (as 381.2 kg and 0.80 g/L for antibody A) ÷ 340 g as above = 89.8%.

In the Parental antibody B case, harvest recovery of parental antibody B = Total protein collected after clarification/Total protein produced in bioreactor = Product of harvest volume and harvest titer (as 350.1 kg and 0.65 g/L for antibody B) ÷ 256 g as above = 88.7%.

The parental antibodies had excellent purity and monodispersity as shown in Table 2. Each of the parental antibodies showed low level of impurities and aggregate levels, as well as low residual process-related impurities after primary purification. Taken together, the entire two processes for antibody manufacturing were compatible for larger scale manufacturing.

**TABLE 2:** Summary of parental antibody purity and process-related impurities after primary purification.

Parental antibody	Purity by SEC-HPLC (%)	Charge variants by CEX-HPLC			Residual HCP (%)	Residual protein A (%)	Residual HCD	Endotoxin	Bioburden (mL)
		Acidic peak	Main peak (%)	Basic peak (%)					
A	98.8	20.10%	68.10	11.80	0.0042	0.0005	44 pg/mg	0.06 EU/mg	<1 CFU/10
B	98.4	17.00	64.40	18.60	<0.0008	0.0003	<0.4 pg/mg	0.06 EU/mg	<1 CFU/10

Note: SEC-HPLC, size exclusion chromatography high performance liquid chromatography; CEX, cation exchange; HCP, host cell protein; HCD, host cell DNA; CFU/10 mL, colony-forming unit per 10 mL sample.

The sample purity by SEC-HPLC, was tested by the SEC-HPLC, method, charge variants of sample was tested by the CEX-HPLC, method, residual HCP, residual Protein A, residual HCD, endotoxin, Bioburden of samples were tested by specific methods which were all outlined in the main body of this paper.

### 3.2 Process optimization of controlled fab-arm exchange reaction

Several reducing agents such as cysteamine hydrochloride (2-MEA) (Labrijn et al., 2013), dithiothreitol (DTT) (Goulet et al., 2018), and glutathione (GSH) (Rispens et al., 2011; Shatz et al., 2016) have been reported to catalyze the cFAE reaction. Since the reductant DTT may not be stable enough for pilot manufacturing process and 15 mM GSH resulted in cFAE recoveries between 68 and 88%, we focused on using 2-MEA for more efficient formation of target bsAb. This study utilized 2-MEA as a catalyst to mediate cFAE reaction process in varying conditions of reaction pH, temperature, diafiltration, and reduction time. Protein samples from a bench-scale bioreactor were used for cFAE reaction process optimization.

#### 3.2.1 Effect of reaction pH on the cFAE reaction

**FIGURE 1:**
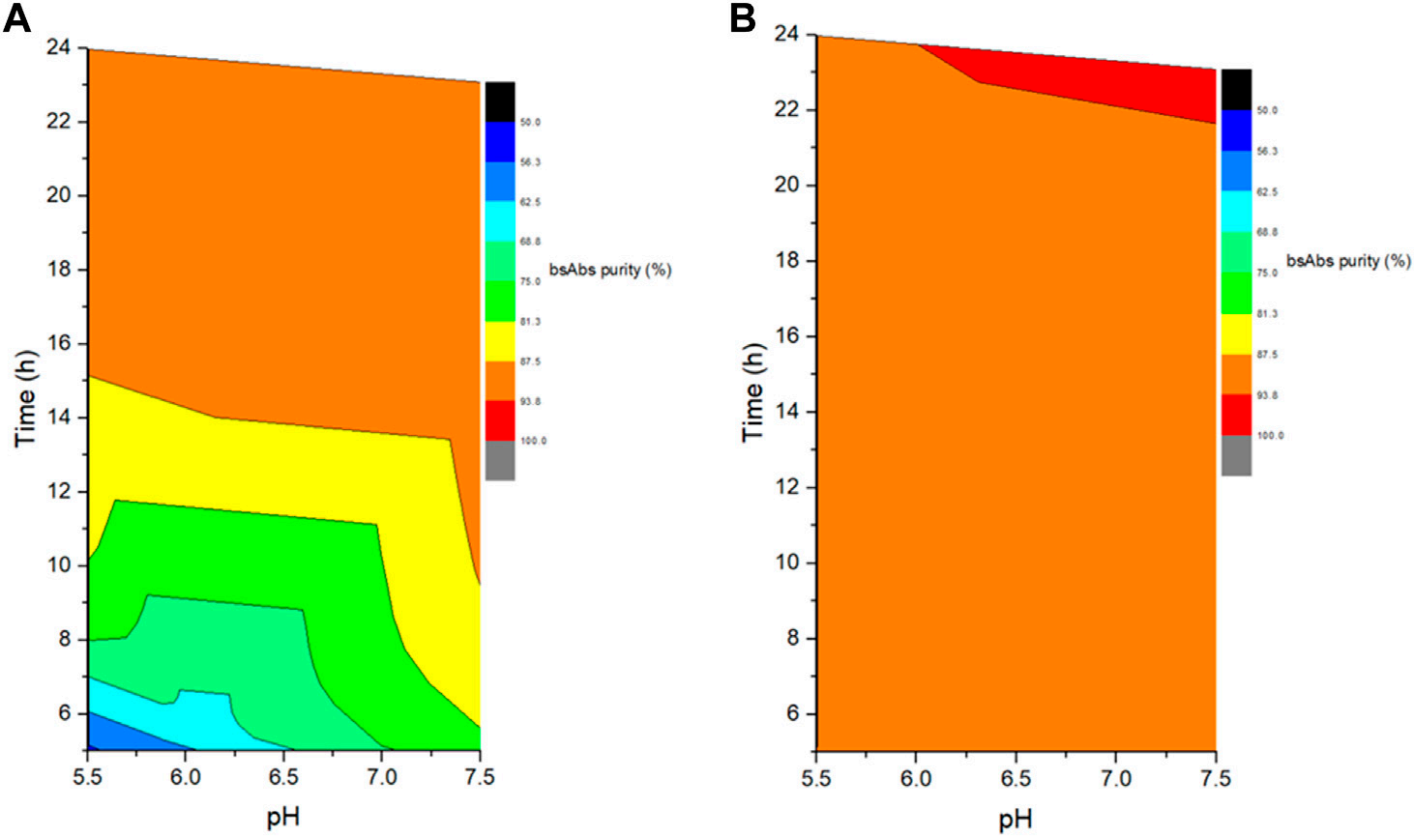
Effect of different reaction pH and time on the exchanged products. **(A)** The left figure indicated the cFAE reaction at 18°C; **(B)**. The right figure indicated the cFAE reaction at 26°C.

The role of reaction pH was studied by performing cFAE in buffer pH 5.5 or 7.5 (with control range of ±0.2, similarly hereinafter). An equimolar amount of parental antibody A and B from bench-scale bioreactors were used for cFAE reaction pH study. The samples were divided into two portions and placed in water bath at 18°C and 26°C, respectively. Sampling was performed at reaction timepoints of 5, 8, 12, and 24 h in both reaction temperature conditions, followed by a diafiltration step to remove the reducing agent, and then incubated at room temperature for oxidation of the hinge disulfide groups. The bsAb critical quality attributes of these retained samples were then analyzed by CE-NR, SEC-HPLC, and CEX-HPLC. After the screening reactions, the bsAb formation efficiency and CE-NR purity of bsAbs under pH 7.5 reaction conditions were clearly higher than those under pH 5.5 reaction conditions at both reaction temperatures (18°C and 26°C) (Figure 1). Therefore, the reduction reaction was set to be run at pH 7.5.

#### 3.2.2 Effect of reaction temperature on the cFAE reaction

**FIGURE 2:**
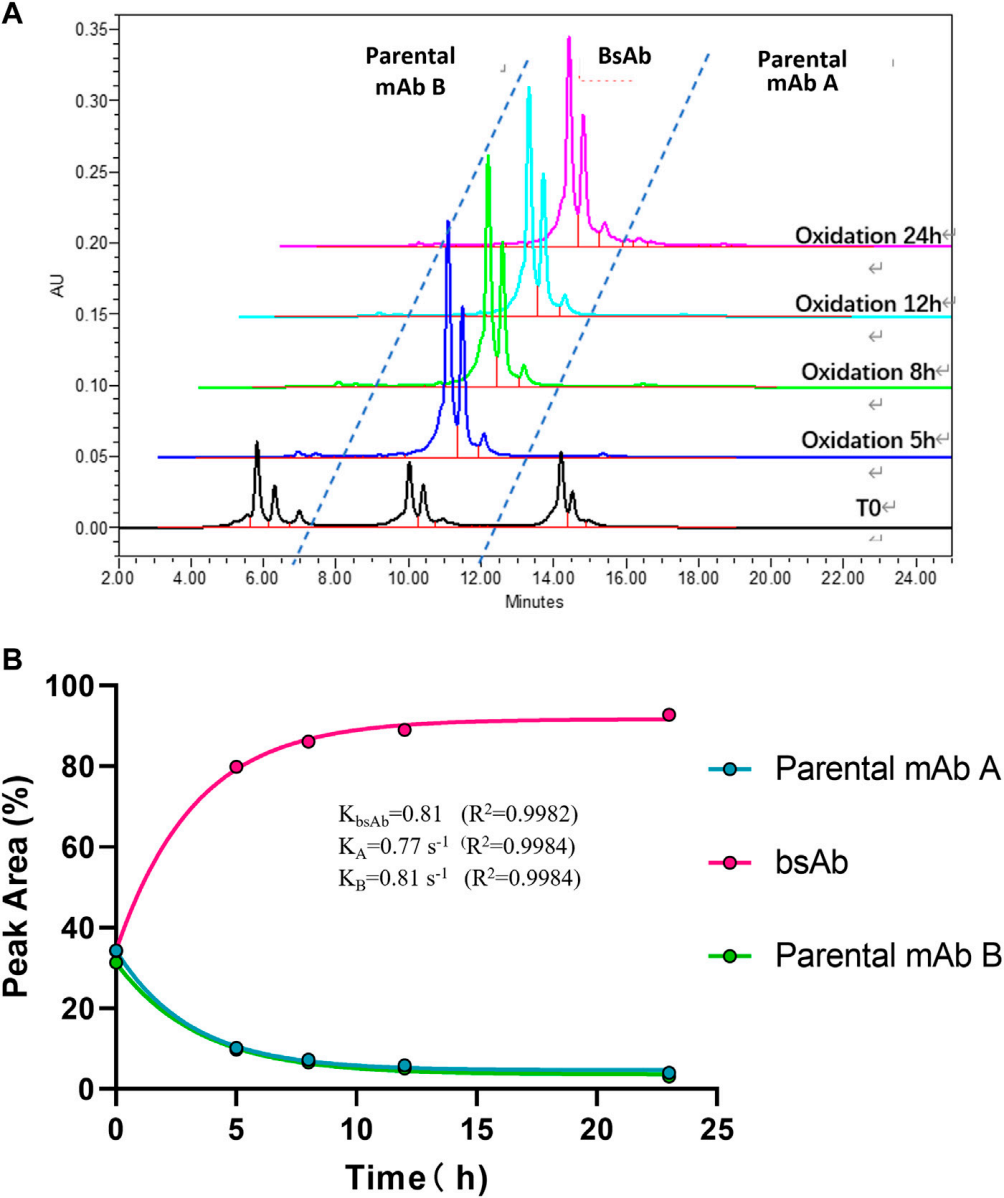
CEX-HPLC analysis for bispecific antibody formed under the condition of 18°C and pH 7.5. **(A)** CEX-HPLC data for parental antibodies and bsAb. The figure indicated the integration values versus time for each parental antibody. **(B)** Statistic analysis of bsAb association and parental mAbs dissociation. The rate constants for association and dissociation of bsAb formation were determined using a single exponential decay fit. R2 is a goodness-of-fit measure for curve fitting. Curve fitting and figure was generated by graphpad prism.

**FIGURE 3:**
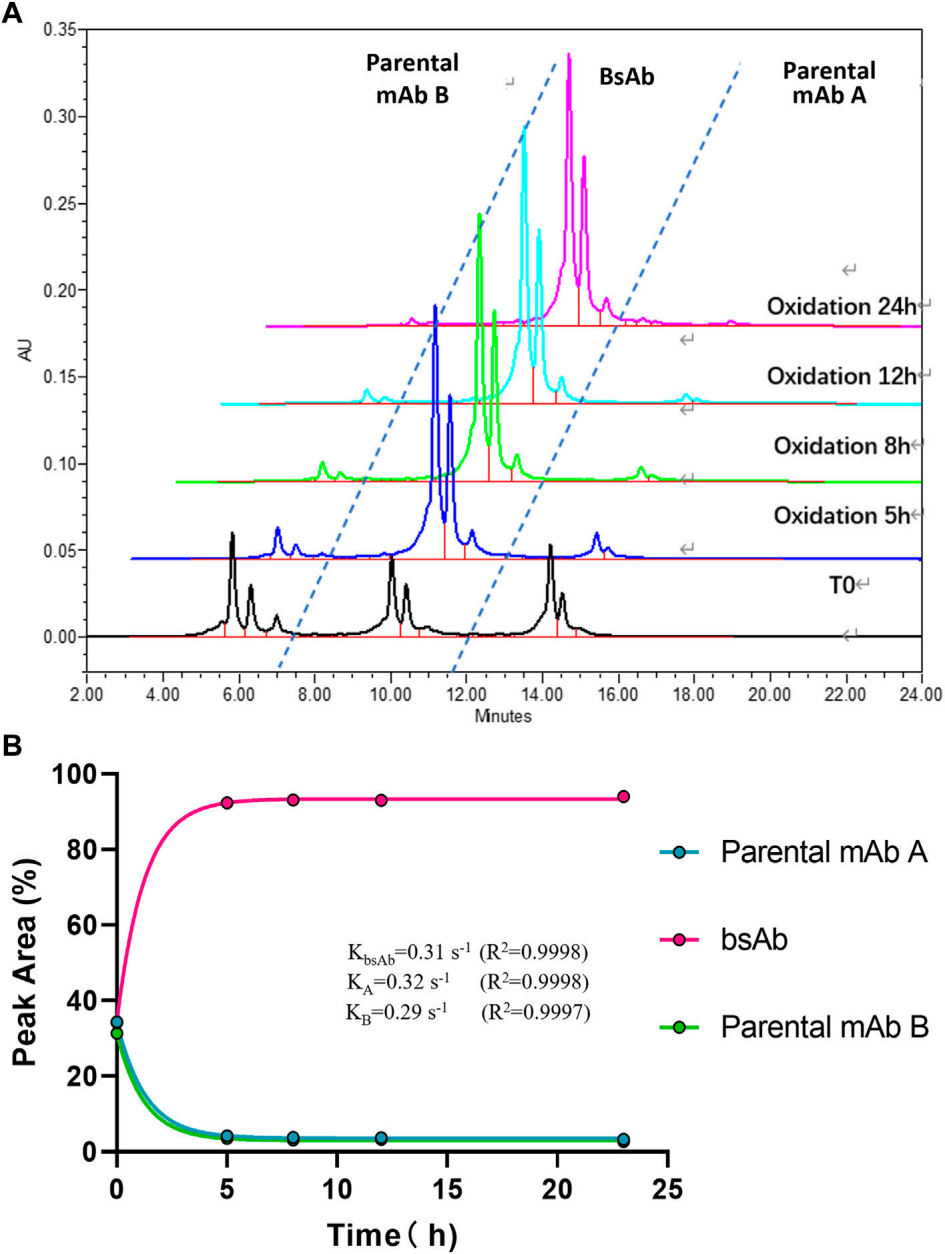
CEX-HPLC analysis for bispecific antibody formed under the condition of 26°C and pH 7.5. **(A)** CEX-HPLC data for parental antibodies and bsAb. The figure indicated the integration values versus time for each parental antibody. **(B)** Statistic analysis of bsAb association and parental mAbs dissociation. The rate constants for association and dissociation of bsAb formation were determined using a single exponential decay fit. Curve fitting and figure was generated by graphpad prism.

To optimize the cFAE reaction conditions, two different temperatures, 18°C and 26°C were evaluated in a fixed pH of 7.5. The bsAb purity as analyzed by CEX-HPLC was much higher than the other reaction timepoints when the reaction time was 8 and 12 h both at 18°C and 26°C (Figures 2, 3; Supplementary Table S-1 for more analytical data). The bsAb formation involved association and dissociation of parental A and B antibodies. Changing the temperature affected both the dissociation rate constant of the parental antibodies during reduction as well as the bsAb association and oxidation. However, the rate constant of bsAb association at 26°C was much higher than that at 18°C, indicating that higher temperature could facilitate better bsAb formation. Considering the processing time of subsequent ultrafiltration and buffer exchange, final reduction reaction process conditions were determined as 8–10 h at a GMP facility room temperature environment of 18∼26°C with pH 7.5 buffer condition.

#### 3.2.3 Effect of diafiltration process on the 2-MEA removal

**FIGURE 4:**
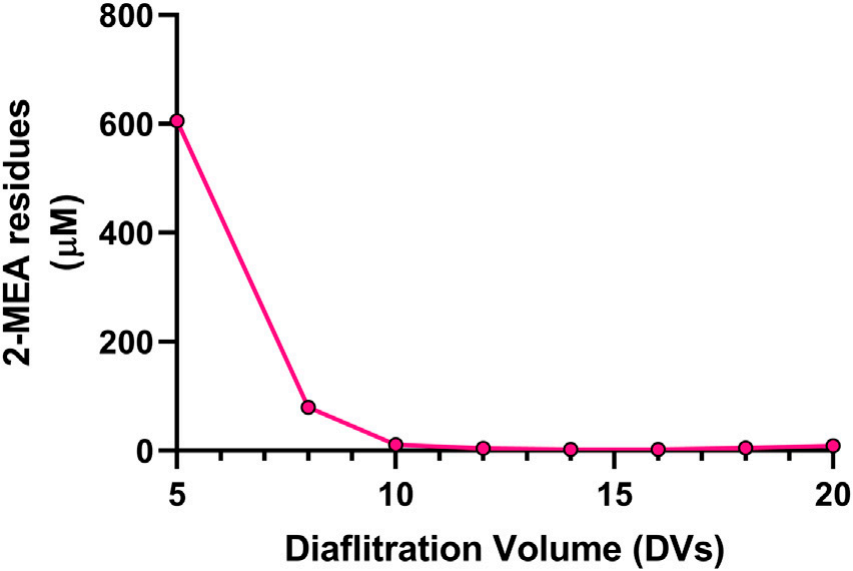
Effect of diafiltration process on the 2-MEA removal. Y axis indicated 2-MEA residual concentrations and X axis means different buffer exchange times.

A critical final step in the cFAE process is the removal of the reductant, since the incomplete removal of 2-MEA may lead to partial reoxidation of the parental antibodies and bsAb, resulting in poor yield in the generation of the bsAbs as well as having poor quality of target bsAbs that could interfere with functional performance. To address these potential issues, we optimized the diafiltration process to remove 2-MEA. Residual 2-MEAs were assessed across different diafiltration volumes during the cFAE process, as the results were shown in Figure 4. The optimal conditions, based on identified diafiltration membrane (Millipore Biomax PES, 30 kDa molecular weight cut-off), an inlet flow rate of 271 L per square meter per hour (LMH) transmembrane pressure (TMP) of 0.7 bar and ≥10 diafiltration volumes, resulted in a comparable low level of residual 2-MEA and end-product quality without loss of bsAb quality (outlined in Supplementary Table S-2 for size purity and charge variants data).

#### 3.2.4 Effect of oxidation time on the cFAE reaction

**FIGURE 5:**
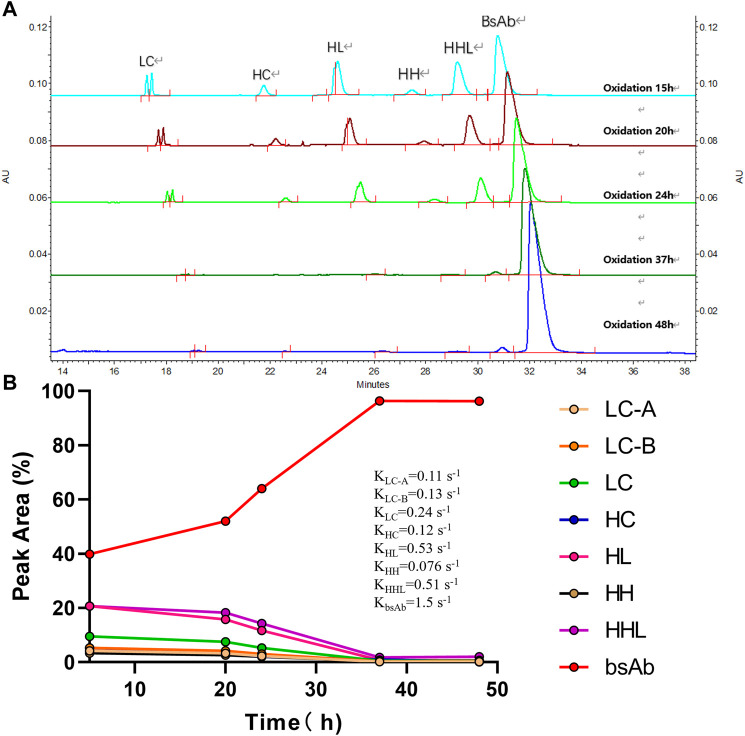
Effect of oxidation time on the cFAE reaction. **(A)** Overlay of CE-NR at different oxidation time. LC contains LC-A and LC-B, separated from parental mAb A and B. HC contains HC-A and HC-B, separated from parental mAb A and B, the molecular weights of HC-A and HC-B are similar and cannot be distinguished from the CE-SDS spectrum. HL contains HL-A and HL-B, separated from parental mAb A and B. **(B)** The integration values for each peak versus oxidation time were shown. The rate constants for association and dissociation of bsAb formation were determined using a linear interpolation fit. For simplicity, slope was recognized as rate constant. Curve fitting and figure was generated by graphpad prism.

Besides 2-MEA removal, oxidation is another important factor for bsAb formation since oxidation of the bsAbs is required to form hinge disulfide-stabilized structures. The oxidation time was screened for the generation of higher yield bsAbs with high purity. The purity results of produced bsAb across different oxidation times were shown in Table 3; Figure 5. The CE-NR analysis showed the purity of produced bsAb could reach 96.3% at room temperature with surface inlet air and continuous stirring after a 48-h reaction. Furthermore, all the purity results of tested bsAbs from SEC-HPLC across different oxidation times were above 98%, and the purity of CE-NR improved with the extension of reaction time, as low molecular weight species reduced increasingly with longer reaction time. In addition, the efficiency of the produced bsAb was evaluated by the rate constant, which was calculated from a linear interpolation fit curve. The migration time of the CE-SDS peaks in Figure 5A drifted as the injection sequence proceeded. Multiple factors could induce the migration time drift in the CE system (Yan et al., 2019). One possible factor was gel buffer evaporation, which may have resulted in higher relative viscosity and reduced electrophoretic mobility. Another factor that could cause baseline drift is the capillary surface property change. Generally, a drift of within 2 min were deemed to be acceptable in using CE-SDS analytical method development. In Figure 5A, the result showed that the drift of the peak migration time [maximum drift time between the upper and bottom sample was 1.08 min (30.500-29.417 = 1.08)], which is within the method variation value of 2 min. As depicted in Figure 5, bsAb formation was at least 3 times higher than the other parental mAbs. In conclusion, the oxidation time of cFAE reaction in our work was determined to be 48 h.

**TABLE 3:** Quality results under different oxidation time.

Oxidation time (hr)	Purity by SEC-HPLC			Purity by CE-NR		
	HMWS (%)	Monomer (%)	LMWS (%)	Main peak (%)	LMWS (%)	HMWS
15	1.2	98.7	0.1	39.9	60.1	N/A
20	1.2	98.7	0.1	52.0	48.1	N/A
24	1.2	98.7	0.1	64.0	36.0	N/A
37	1.1	98.8	0.1	96.4	3.5	N/A
48	1.2	98.7	0.1	96.3	3.8	N/A

Note: hrs, hours; HMWS, high molecular weight species; LMWS, low molecular weight species; CE-NR, non-reduced capillary electrophoresis, N/A, not applicable.

### 3.3 2-MEA material control study

2-MEA, a mild reducing agent, which could efficiently reduce the IgG1 hinge (Gramer et al., 2013). Since this is a critical component for the bsAb cFAE process, several quality control strategies to ascertain the raw material control of 2-MEA, in terms of in-process monitoring and impurity residual level.

#### 3.3.1 Raw material control

Before entering the cFAE reaction, the starting material 2-MEA was fully analyzed by the manufacturers to meet GMP compliance terms of identification test by IR spectrum, metal trace analysis, and cyclic voltammetry testing. The intensity peak at IR spectrum peaks correspond to C-H, N-H and R-S-H stretch of 2-MEA (Table 4; Supplementary Figure S1) (Mohrig et al., 2010; Mohamed et al., 2017; Tomar et al., 2022). The metal trace results in the certificate of analysis of vendor showed a low level of residual metal content and met the specification range (e.g., <5 mg/kg for Aluminum, Barium, Bismuth, etc., analyzed by Sigma). In addition, the cyclic voltammetry testing confirmed the thiol oxidation and reduction potential of 2-MEA from the 0.3 V oxidation peak at 0.3 V (Lin et al., 2013; Silva et al., 2018), and the reduction peak at −0.5 V indicated the oxidation state underwent a reduction reaction on glassy carbon electrode (GCE) (Salmanpoura et al., 2016) (Supplementary Figure S2).

**TABLE 4:** IR peak assignments for tested 2-MEA.

Peak	Frequency (cm)	Assignment
1	3432^–1^	N-H stretch
2	3015^–1^	C-H stretch
3	2502^–1^	S-H stretch
4	1595^–1^	N-H bend
5	1487^–1^	-CH_2_- bend

Note: these frequency values were read from the Infrared Spectroscopy (IR) spectrum as outlined and labeled in Figure 6, and the assignment to each function group was confirmed from referenced book as outlined in main body of this paper.

#### 3.3.2 In-process monitoring

**FIGURE 6:**
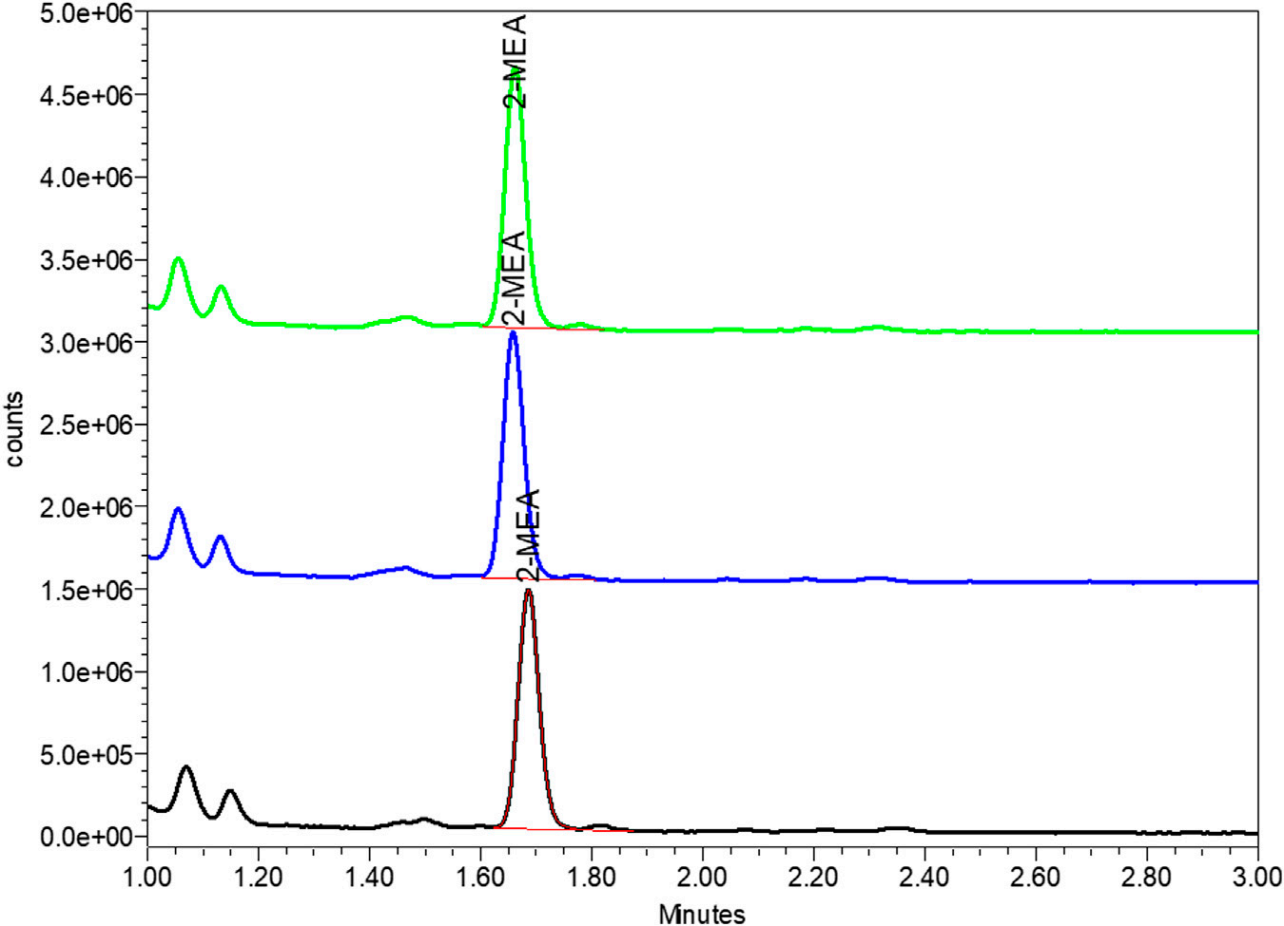
Typical chromatogram of 2-MEA residue of final drug substance by FLD-UPLC. The Y axis represented the signal of tested article, the X axis represented the peak retention time of in UPLC column. Green, blue and red peak represented the tested 2-MEA peak of three different sample batches.

During the pilot scale of bsAb production, the 2-MEA residues across different purification steps were monitored by quantitative High-performance Liquid Chromatography with fluorescence detection method. After a certain analytical development and phase-appropriate qualification work, this study output a good resolution 2-MEA analytical method. Table 5; Figure 6 of typical 2-MEA chromatograms showed that the residual 2-MEA in product can be gradually removed to a certain level by further purification steps.

**TABLE 5:** In-process test results of residual 2-MEA across different purification steps.

Process step	Residual 2-MEA by FLD-UPLC (μM)
*In-vitro* assembly	39.9
Cation exchange chromatography	3.7
Anion exchange chromatography	3.8
Ultrafiltration/diafiltration	5.4
Drug substance	5.1

Note: 2-MEA, cysteamine hydrochloride; μM, μmol/L; FLD-UPLC, fluorescence detector based ultra-performance liquid chromatography (used hereinafter).

### 3.4 Process scale-up study of controlled fab-arm exchange reaction

The optimized conditions were subsequently scaled up to a larger scale, using 3.2 g/L of each parental antibody in buffer pH 7.5 to create a 57.8 L pilot batch. The starting material of parental antibodies for the cFAE reaction were generated from the 200-L manufacturing process. The comparison of cFAE process parameters between small and large scale was shown in Table 6.

**TABLE 6:** Process parameters of cFAE at different scales.

Process parameter	Reaction scale	
	620 mL	57.8 L
Parental mAb A concentration by UV	3.2 g/L	3.2 g/L
Parental mAb B concentration by UV	3.2 g/L	3.2 g/L
Input Parental mAb A or B	2.5 g	183.6 g
2-MEA conc.by FLD-UPLC	75 mM	75 mM
Reaction pH	7.3∼7.7	7.3∼7.7
Reduction Time	8 h	9 h
Reaction temperature	18°C–26°C	18°C–26°C
2-MEA removal procedure	Diafiltration	Diafiltration

Note: mM, mmol/L; conc., concentration.

Further process and quality analysis of the cFAE reaction between scales were summarized in below Table 7. The calculated exchange yield of pilot scale was 99.3%, which was in close level agreement to previous results using much small volume conditions (Labrijn et al., 2013). Purity by CE-NR analysis demonstrated complete re-oxidation of the hinge disulfide groups by the end of cFAE. The SEC-HPLC purity of the reaction product showed that the process did not induce in product aggregation (>98.8% monomer).

**TABLE 7:** Exchange yield, charge variants, purity and residual 2-MEA following cFAE reaction.

Scale	Actual formed bsAb (g)	Exchange yield^a^	Charge variants by CEX-HPLC			Purity by SEC-HPLC (%)	Purity by CE-NR (%)	Residual 2-MEA by FLD-UPLC
			Acidic peak (%)	Main peak (%)	Basic peak (%)			
620 mL	4.5	89.4%	19.3	53.8	26.9	98.6	96.0	Not detected
57.8 L	367.2	99.3%	16.9	61.7	21.4	98.8	96.8	19.6 μM

Note:

^a^ The exchange yield of cFAE, was defined as the conversion ratio of parental mAb A. Theoretical molecular weight of bsAb: 145.5 kDa; Molecular weight of parental mAb A: 144.5 kDa.

Exchange yield of cFAE $=Actual\;consumption\;protein\;amount\;of\;Parental\;mAb\;A÷Input\;protein\;amount\;of\;Parental\;mAb\;A=Molar\;conc.\;of\;formed\;bsAb\left(=2\times Molar\;conc.\;of\;Parental\;mAb\;A\right)\times Molar\;weight\;of\;Parental\;mAb\;A÷Input\;amount\;of\;Parental\;mAb\;A$ .

In the 620 mL-scale case, the exchange yield was calculated as (4.5 g bsAb ÷ 145.5 kDa × $\frac{1}{2}$ × 144.5 kDa) ÷2.5×100%= 89.4%;

In the 57.8 L-scale case the exchange yield was calculated as (367.2 g bsAb $÷145.5\text{ kDa}\times \frac{1}{2}\times 144.5\text{ kDa}$ ) ÷183.6 g=99.3% .

The binding activity of produced bsAb was assessed by ELISA binding (Supplementary Table S-4; Figure 3). These results showed that the large-scale produced bsAb showed comparable binding properties between batches. Next, mass spectroscopy analyses on the deglycosylated intact Parental mAb A, B and bispecific antibody AB were performed to confirm the molecular integrity (Supplementary Figure S4). For prepared bsAb, a measured mass of 145,535 Da was analyzed for production batch, which agreed with the theoretical masses for the assembled bsAb 145,538 Da, indicating the bsAb were correctly prepared. Upon detailed analysis, no chain mispaired homodimers nor swapping of light chains appeared in as expected (Supplementary Table S-5). The followed stability of the large-scale produced bsAb upon storage in final drug product formulation has been studied, no major changes in product quality over a period of 6 months at 2∼8°C long-term storage (Supplementary Table S-6).

The process was conducted in a controlled environment to minimize any contamination of endotoxin (Supplementary Table S-3). And there were insignificant levels of residual 2-MEA concentrations in final drug substance (Table 5). All these results showed no impact on the purity and impurities of final bsAbs. The 2-MEA mediated cFAE technique for bsAb production performed well among different production scales and could meet manufacturing requirements.

## 4 Discussion

We evaluated the cFAE process parameters of reaction time, pH, temperature, residual reductant removal procedure, and oxidation time. In addition, we also outlined the optimization of cFAE process by varying pH, temperature, time for reduction, oxidation, and diafiltration. The most favorable conditions for the cFAE reaction were achieved at a pH setpoint of 7.5 and a temperature range of 18°C–26°C. The reaction time range of 8–10 h, coupled with optimal UF/DF process followed by an oxidation reaction time of 48 h produced optimal results. The optimized UF/DF process to achieve a lower residual 2-MEA level were recommended to use inlet flow rates of 271 LMH, a TMP of 0.7 bar, and conduct diafiltration with at least 10 volumes, using a Millipore Biomax PES membrane with a molecular weight cut-off of 30 kDa.

**FIGURE 7:**
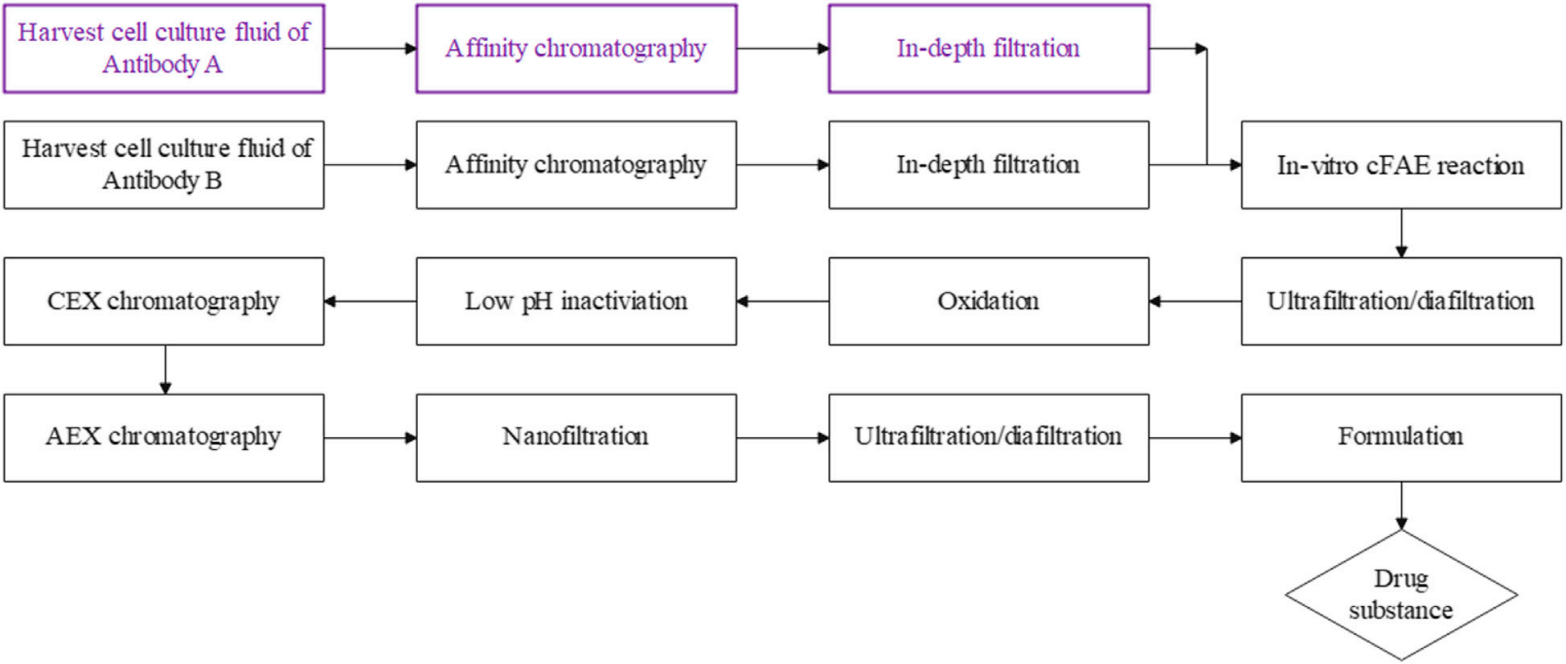
A summary of the cFAE-based bsAb manufacturing process. The parental mAbs were purified using standard protein A chromatography and in-depth filtration, followed by the *in-vitro* cFAE reaction, low pH viral inactivation, cation-exchange and anion-exchange chromatography, nanofiltration, the ultrafiltration/diafiltration and formulation step was employed as the final unit operation to formulate the assembled bispecific antibody into final drug substance.

In our study, the cFAE process was scalable from the 570 mL to 57.8 L scale, resulting in a total 0.4 kg of target bsAb generated in a reaction volume of 57.8 L, with product purity >95% and 90% exchange yield from parental mAbs. The overall established manufacturing process was outlined in Figure 7. We presented the essential requirements for a successful scalable cFAE process that included key process parameters such as: the quality of the parental mAbs, starting and residual control of reducing agent, reaction parameters such as reaction temperature and mixing time, which all affect the process yield. Based on the current manufacturing process, larger scale-up production of bsAbs with comparable quality can be expected. Since the cFAE-based manufacturing process was well-understood and robust, this process could be scalable to the level of thousands of liters.

Based on our comprehensive analytical and residue assessment, the residual reductant 2-MEA in the final target product can be of low risk. The ophthalmic drug of Cysteamine Hydrochloride was approved by US FDA in 2012 with product name as Cytaran^®^, with a daily dose of 7.8 mg/day based on referenced FDA drug approval review document (FDA, 2012). According to the safety assessment data of Table 8, the residual 2-MEA in final dosage is about 41.0 μg, based on the evaluation, the maximum residual 2-MEA in final target product is far below the limit of Cytaran^®^ and is in low risk.

**TABLE 8:** Evaluation on the safety of residual 2-MEA.

Impurity	2-MEA conc. In target drug (μM)	Max. dose of target drug	Max. Clinical dosage of target drug	Estimated 2-MEA level per dose*	Acceptance criteria as Cytaran^®^ a benchmark (mg)	Risk level
2-MEA	5.1	20 mg/kg	1,400 mg^#^	41.0 μg	7.8	Low

Note: # 1,400 mg was calculated by 70 kg (average patient weight) ×20 mg/kg (maximum dose); * Estimated 2−MEA level per dose=2−MEA conc. in target drug×Molar weight of 2−MEA×Total injection volume of target drug . In above case the estimated 2-MEA level per dose was calculated as follows: 5.1 μM × 113.61 g/moL × (1,400 mg ÷ 20 mg/mL) = 41.0 μg.

The supernatant cFAE process was also investigated in our previous studies, 2-MEA was added in the supernatant of harvest cell culture fluid to generate a certain amount of bsAb aim for early experiments directly. Study results showed lower assembly efficiency (data not shown here), unlike the process results of other researchers (Williams et al., 2015; Shatz et al., 2016; Giese et al., 2018) which used glutathione as cFAE reductant. It indicated the reductant type, concentration and process-appropriate parameters could affect the process exchange efficiency.

In conclusion, the cFAE methodology was a scalable process that only required a mild reducing environment followed by reductant removal. This process optimization could be feasibly incorporated into a standard platform antibody manufacturing process. This bsAb production method could achieve higher yields and mitigate potential quality risks than that of other bsAb production methods.

## Figures and Tables

**Table:** 

**AEX**	Anion exchange chromatography
**bsAb**	bispecific antibody
**BCMA**	B cell maturation antigen
**β-ME**	β-Mercaptoethanol
**CD3**	Cluster of differentiation 3
**CE-SDS**	Capillary electrophoresis sodium dodecyl sulfate
**cMET**	cellular-mesenchymal epithelial transition factor
**CEX**	Cation exchange chromatography
**cFAE**	controlled fab-arm exchange
**CHO**	Chinese hamster overlay
**EGFR**	Epidermal growth factor receptor
**FDA**	Food and drug administration
**FLD**	Fluorescence detector
**GCE**	Glassy carbon electrode
**GMP**	Good manufacturing practice
**2-MEA**	Cysteamine hydrochloride
**HCCF**	Harvested cell culture fluid
**HRP**	Horseradish peroxidase
**HCP**	Host cell protein
**HMWS**	High molecular weight species
**I.D.**	Inner diameter
**IR**	Infrared spectroscopy
**LC-MS**	Liquid chromatography mass spectrometry
**LMWS**	Low molecular weight species
**mAb**	monoclonal antibody
**NEM**	N-ethylmaleimide
**PCR**	Polymerase chain reaction
**PES**	Polyether sulfone
**SEC**	Size exclusion chromatography
**TMP**	Trans-membrane pressure drop
**TMB**	3,3,5,5-Tetramethyl benzidine
**UF/DF**	Ultrafiltration and diafiltration
**UV**	Ultraviolet-visible
**VCD**	viable cell density

## Data Availability

The original contributions presented in the study are included in the article/Supplementary Material, further inquiries can be directed to the corresponding author.
