# Supplementary material for: Large scale controlled Fab exchange GMP process to prepare bispecific antibodies
Source: Front Bioeng Biotechnol. 2024 Jan 12;11:1298890. doi: 10.3389/fbioe.2023.1298890 (PMC10812119; doi:10.3389/fbioe.2023.1298890)
Supplement: Supplementary file 1 [file DataSheet1.PDF]

Supplementary Materials

Large Scale Controlled Fab Exchange GMP Process to Prepare Bispecific Antibodies

Xia Yao[1, †], Mingquan Xie[1, †], Yinyin Ben[1], Yixiang Zhu[2], Gaoqiang Yang[2],

Simon Chi Wai Kwong[2], Zhengliang Zhang[2], Mark L. Chiu[1,3]*

[1]Tavotek Biotherapeutics, Xinghu Street, Suzhou Industrial Park, Suzhou, China

[2] Bioworkshops (Suzhou) Limited, Dongwang Road, Suzhou Industrial Park, Suzhou, China

[3] Tavotek Biotherapeutics, 727 Norristown Road, 3 Spring House Innovation Park, Suite 100, Lower Gwynedd, PA, USA

*** Correspondence:** Mark L. Chiu mark.chiu@tavotek.com

[†] These authors contributed equally to this work.

1 Supplementary Data

Not applicable.

2 Supplementary Figures and Tables

2.1 Supplementary Tables

Supplementary Table S-1.

Ab quality under different reduction reaction conditions

pH	Temperature	Time	SEC-HPLC			CEX-HPLC			CE-NR		
			HMWS	Monomer	LMWS	Parental mAb A	BsAb	Parental mAb B	Main peak	LMWS	HMWS
5.5	N/A	0	7.7%	92.0%	0.3%	47.5%	8.3%	44.2%	93.6%	6.0%	0.5%
5.5	18℃	5 h	10.8%	86.6%	2.7%	22.1%	55.0%	23.0%	81.1%	17.8%	1.2%
		8 h	7.1%	92.7%	0.2%	12.9%	74.9%	12.2%	91.5%	8.2%	0.2%
		12 h	7.2%	92.6%	0.2%	8.1%	84.0%	8.0%	93.2%	6.7%	0.2%
		24 h	7.2%	92.6%	0.2%	4.1%	92.2%	3.7%	91.7%	8.0%	0.3%
	26℃	5 h	7.3%	92.4%	0.3%	5.3%	88.6%	6.0%	95.4%	4.2%	0.4%
		8 h	7.0%	92.7%	0.2%	3.6%	92.8%	3.6%	91.4%	8.6%	N/A
		12 h	7.1%	92.6%	0.2%	3.4%	93.4%	3.2%	93.7%	6.2%	0.2%
		24 h	7.0%	92.7%	0.2%	3.2%	93.7%	3.0%	92.0%	7.7%	0.3%
7.5	N/A	0	9.2%	90.6%	0.2%	34.4%	34.2%	31.4%	93.6%	6.0%	0.4%
7.5	18℃	5 h	7.4%	92.5%	0.2%	10.2%	79.9%	9.8%	93.1%	6.4%	0.4%
		8 h	7.0%	92.9%	0.1%	7.3%	86.1%	6.6%	95.8%	3.9%	0.3%
		12 h	6.9%	92.9%	0.1%	5.9%	89.0%	5.1%	96.1%	3.5%	0.2%
		24 h	6.6%	93.2%	0.1%	4.0%	92.8%	3.1%	93.2%	6.6%	0.3%
	26℃	5 h	6.6%	93.3%	0.1%	4.2%	92.4%	3.5%	89.4%	10.4%	0.2%
		8 h	6.9%	93.0%	0.1%	3.8%	93.1%	3.1%	96.0%	3.8%	0.2%
		12 h	7.0%	92.9%	0.1%	3.7%	93.0%	3.3%	96.2%	3.6%	0.2%
		24 h	6.6%	93.3%	0.1%	3.3%	94.0%	2.7%	91.0%	8.7%	0.2%

N/A: not applicable; HWMS: high molecular weight species; LWMS: low molecular weight species, mAb: monoclonal antibody; CE-NR: non-reduced SDS - capillary electrophoresis; CEX-HPLC: cation exchange high performance liquid chromatography; SEC-HPLC: size exclusion chromatography – high performance liquid chromatography.

Supplementary Table S-2.

Residual 2-MEA and Ab quality at different buffer change times

Diafiltration Volume	SEC-HPLC			CEX-HPLC			CE-NR			2-MEA
	HMWS	Monomer	LMWS	Parental B mAb	bsAb	Parental A mAb	Main peak	LMWS	HMWS	
5	/	/	/	/	/	/	/	/	/	605.7 μM
8	/	/	/	/	/	/	/	/	/	79.6 μM
10	1.1%	98.8%	0.1%	2.3%	95.4%	2.3%	96.1%	3.9%	0	11.0 μM
12	1.1%	98.7%	0.1%	2.3%	95.5%	2.2%	95.0%	5.0%	0	4.1 μM
14	/	/	/	/	/	/	/	/	/	2.4 μM
16	/	/	/	/	/	/	/	/	/	2.0 μM
18	/	/	/	/	/	/	/	/	/	5.2 μM
20	1.1%	98.7%	0.2%	2.4%	95.1%	2.5%	95.8%	4.2%	0	9.2 μM

Note: HWMS: high molecular weight species; LWMS: low molecular weight species; mAb: monoclonal antibody; CE-NR: non-reduced SDS - capillary electrophoresis; CEX-HPLC: cation exchange high performance liquid chromatography; SEC-HPLC: size exclusion chromatography – high performance liquid chromatography.

Supplementary Table S-3.

Ab Quality before and after controlled Fab-arm Exchange reactions

Test Items		Parental mAb A		Parental mAb B		bsAb		Final drug substance	
		**15 L**	**200 L**	**15 L**	**200 L**	**15 L**	**200 L**	**15 L**	**200 L**
SEC-HPLC	Monomer	99.0%	98.8%	99.0%	98.4%	98.7%	98.8%	99.0%	98.8%
	HMWS	0.9%	1.2%	1.0%	1.6%	1.2%	1.0%	1.0%	1.2%
	LMWS	0.0%	0.0%	0.0%	0.0%	0.1%	0.1%	0.0%	n.d.
NR-CE-SDS	Main peak	97.8%	98.6%	96.2%	97.5%	96.3%	96.8%	96.3%	97.0%
	HMWS	2.1%	n.d.	3.5%	n.d.	3.8%	3.2%	3.7%	2.8%
	LMWS	0.0%	1.4%	0.3%	2.5%	0.0%	n.d.	0.0%	n.d.
R CE-SDS	LC+HC	98.9%	98.7%	98.5%	98.6%	98.6%	98.5%	97.6%	98.5%
	NGHC	0.3%	0.5%	0.4%	0.4%	0.5%	0.5%	0.5%	0.5%
	Others	0.8%	n.d.	1.0%	n.d.	1.0%	n.d.	1.9%	n.d.
CEX-HPLC	Acid peak	20.5%	20.1%	19.5%	17.0%	18.8%	16.9%	19.0%	17.0%
	Main peak	60.6%	68.1%	61.1%	64.4%	54.3%	61.7%	55.9%	63.0%
	Basic peak	18.9%	11.8%	19.3%	18.6%	26.9%	21.4%	25.1%	20.0%
Host cell protein		< 0.0049%	0.0042%	< 0.0043%	<0.0008%	/	0.0008%	0.00003%	<0.00008%
Residual protein A		0.0006%	0.0005%	0.00005%	0.0003%	/	0.0004%	< 00004%	<0.00004%
Host cell DNA		25.1 pg/mg	44 pg/mg	< 0.3 pg/mg	<0.4 pg/mg	/	3 pg/mg	0.2 pg/mg	< 0.2 pg/mg
Endotoxin		/	0.06 EU/mg	/	0.06 EU/mg	/	0.13 EU/mg	/	< 0.01 EU/mg
Bioburden		/	<1 CFU/10 mL	/	<1 CFU/10 mL	/	<1 CFU/10 mL	/	<1 CFU/10 mL

NGHC: Non-glycosylated heavy chain; n.d. not detected; L – liter; CFU: colony forming units; HWMS: high molecular weight species; LWMS: low molecular weight species; mAb: monoclonal antibody; NR-CE-SDS: non-reduced SDS - capillary electrophoresis; R-CE-SDS: reduced SDS - capillary electrophoresis; CEX-HPLC: cation exchange high performance liquid chromatography; SEC-HPLC: size exclusion chromatography – high performance liquid chromatography; DNA: deoxyribonucleic acid; EU/mg: endotoxin units per mg; CFU/10 mL: colony forming units per 10 milliliter; LC : light chain; HC: heavy chain; bsAb: bispecific antibody

Supplementary Table S-4.

Functional ELISA activity of bispecific antibody

Testing Items	Batch No.	
	S210723	S210724
Binding activity with antigen A	108%	112%
Binding activity with antigen B	102%	102%

Note: ELISA, enzyme linked immunosorbent assay. Product bioactivity shall not exceed 60-140% relative to the reference.

Supplementary Table S-5.

Molecular weight of parental antibody A, B, and bispecific antibody

Sample name		Deglycosylated intact mass (Da)			
		Modification type	Theoretical mass (Da)	Experimental mass (Da)	Difference* (ppm)
Parental antibody A		Intact (2*Deglycosylation, 2*K loss)	144518.4	144514.5	-26.9
		Intact (2*Deglycosylation, 1*K loss)	144646.5	144645.5	-7.4
Parental antibody B		Intact (2*Deglycosylation, 2*K loss)	146558.6	146555.1	-24.4
		Intact (2*Deglycosylation, 1*K loss)	146686.8	146683.0	-26.0
		Intact (2*Deglycosylation)	146815.0	146813.6	-9.0
Bispecific antibody	**Desired product**	**Intact (2*Deglycosylation, 2*K loss)**	**145538.5**	**145535.7**	**-19.1**
		**Intact (2*Deglycosylation, 1*K loss)**	**145666.7**	**145663.6**	**-21.2**
	Mispaired products	HC-A, LC-B&HC-B, LC-B (2*Deglycosylation, 2*K loss)	146232.3	Not detected	Not applicable
		HC-A, LC-B&HC-A, LC-B (2*Deglycosylation, 2*K loss)	145906.0	Not detected	Not applicable
		HC-A, LC-B&HC-A, LC-A (2*Deglycosylation, 2*K loss)	145212.2	Not detected	Not applicable
		HC-A, LC-A&HC-B, LC-A (2*Deglycosylation, 2*K loss)	144844.7	Not detected	Not applicable
		HC-B, LC-B&HC-B, LC-A (2*Deglycosylation, 2*K loss)	145864.8	Not detected	Not applicable
		HC-B, LC-A&HC-B, LC-A (2*Deglycosylation, 2*K loss)	145171.0	Not detected	Not applicable

Note: HC means heavy chain; LC means light chain; K, lysine; Da, dalton; ppm, parts per million. Text in bold indicated the desired bispecific antibody to be verified.

* The variation of the mass spectroscopy method shall not exceed 100 ppm.

Supplementary Table S-6.

Long-term stability data of representative bispecific antibody (2 – 8 ℃)

No.	Test Items	Acceptance Criteria	Timepoints (Months)			
			**0**	**1**	**3**	**6**
1	Appearance	Colorless to light yellow liquid	Colorless liquid	Colorless liquid	Colorless liquid	Colorless liquid
2	Clarity	≤Reference III	Conforms	Conforms	Conforms	Conforms
3	pH	5.7–6.3	6.1	6.1	6.1	5.9
4	Osmolality	200–300 mOsmol/kg	250	N/A	N/A	N/A
5	Visible particulates	Conforms	Conforms	Conforms	Conforms	Conforms
6	Particulate matter	≥10 µm: NMT 6000 per vial.<br>≥25 µm: NMT 600 per vial;	28<br>4	N/A	N/A	N/A
7	Deliverable volume	≥4.0 mL	4.3	N/A	N/A	N/A
8	Protein	18.0–22.0 mg/mL	20.0	20.0	19.8	20.1
9	PS20 content	0.10–0.30 mg/mL	0.20	N/A	N/A	0.21
10	Isoelectric point (icIEF)	pI should be 8.5–8.9, profile should be consistent with Reference	pI:8.7, others conform	pI:8.7, others conform	pI:8.7, others conform	pI:8.7, others conform
11	Purity (NR CE-SDS)	Main peak% ≥90.0%	97.3%	97.2%	97.4%	97.6%
		Fragments% ≤10.0%	2.7%	2.8%	2.6%	2.4%
12	Purity (R CE-SDS)	2(LCs + HCs) % ≥90.0%	98.3%	98.1%	98.1%	98.3%
		NGHC% ≤5.0%	0.5%	0.5%	0.5%	0.5%
13	Purity (SEC-HPLC)	Monomer% ≥92.0%	98.8%	98.6%	98.4%	98.2%
		Aggregates% ≤5.0%	1.2%	1.4%	1.5%	1.7%
14	Charge variants (CEX-HPLC)	Report acidic peaks area%	17.3%	18.1%	18.8%	19.7%
		Report main peak area%	63.1%	63.3%	62.8%	61.5%
		Report basic peaks area%	19.5%	18.6%	18.4%	18.9%
15	Binding activity (Target A, ELISA)	60%–140% relative to Reference	99%	102%	110%	108%
16	Binding activity (Target B, ELISA)	60%–140% relative to Reference	95%	102%	102%	99%
17	Bacterial endotoxins	<0.25 EU/mg	< 0.01	N/A	N/A	N/A
18	Sterility	No microbial growth detected	No microbial growth detected	N/A	N/A	N/A

Note: N/A: not applicable; NGHC: Non-glycosylated heavy chain; L – liter; HWMS: high molecular weight species; LWMS: low molecular weight species; NR-CE-SDS: non-reduced SDS - capillary electrophoresis; R-CE-SDS: reduced SDS - capillary electrophoresis; CEX-HPLC: cation exchange high performance liquid chromatography; SEC-HPLC: size exclusion chromatography – high performance liquid chromatography; EU/mg: endotoxin units per mg; LC: light chain; HC: heavy chain; bsAb: bispecific antibody; PS20: Polysorbate 20; NMT: not more than.

2.2 Supplementary Figures

A

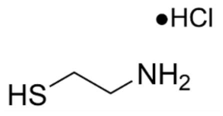

B

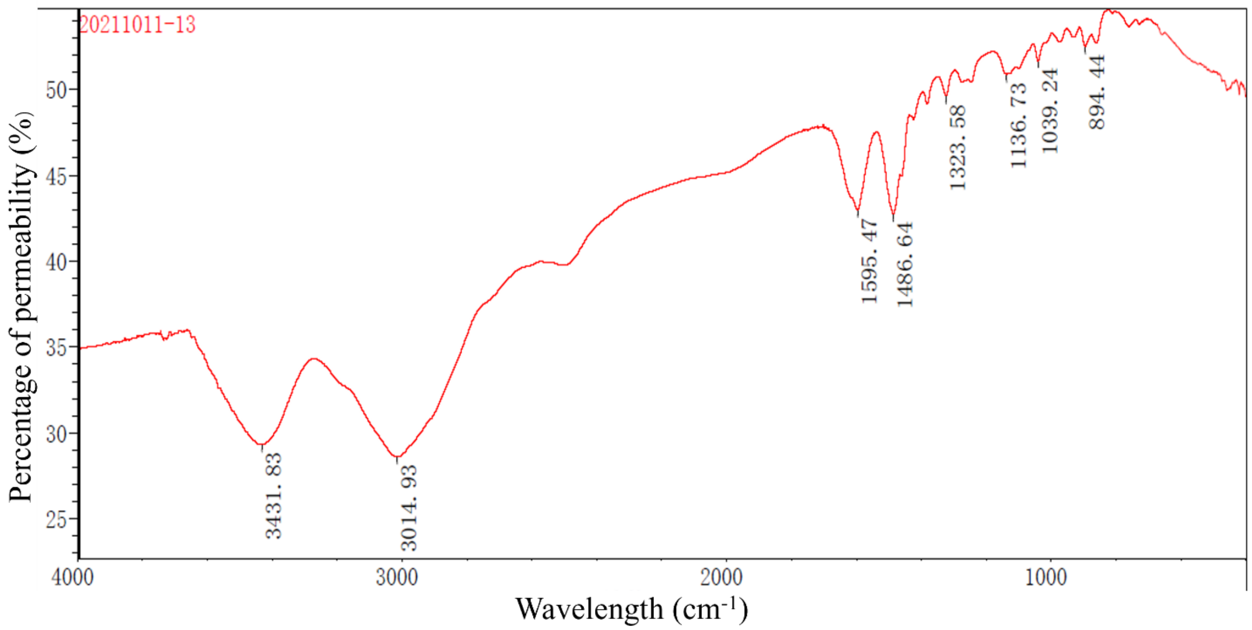

Supplementary Figure 1. Raw material control of 2-MEA by infrared spectroscopy (IR) spectrum. (A) Chemical structure of 2-MEA, it is a form of hydrochloride. (B) IR spectrum of 2-MEA. X axis indicated the wavenumber values (cm^{-1}) while Y axis means the percentage of permeability.

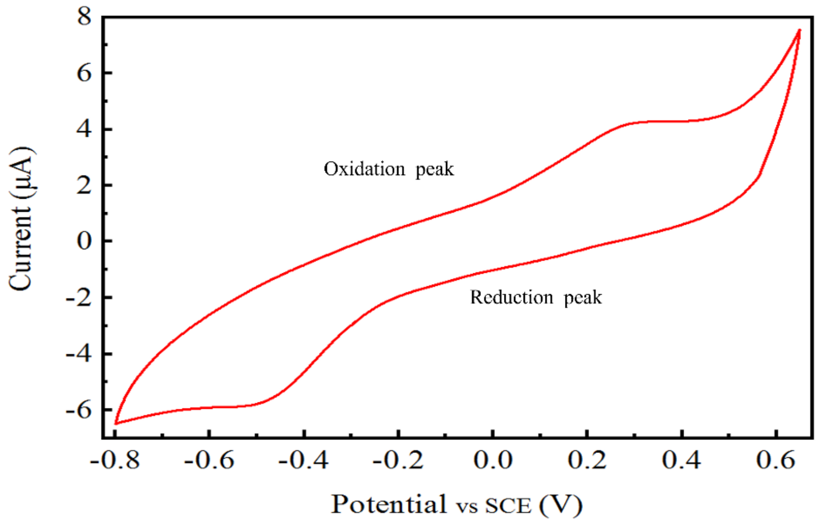

Supplementary Figure 2. Cyclic voltammetry testing of 2-MEA. The Y axis represented the potential relative to the SCE (saturated calomel electrode), and the X axis represented the current intensity at the corresponding potential. The oxidation peak at 0.3 V indicated 2-MEA undergo oxidation reaction on the surface of electrode to generate cysteamine. The reduction peak at -0.5 V indicated the oxidation state undergo a reduction reaction on Glassy carbon electrode (GCE), and the S-S bond breaks to regenerate 2-MEA.

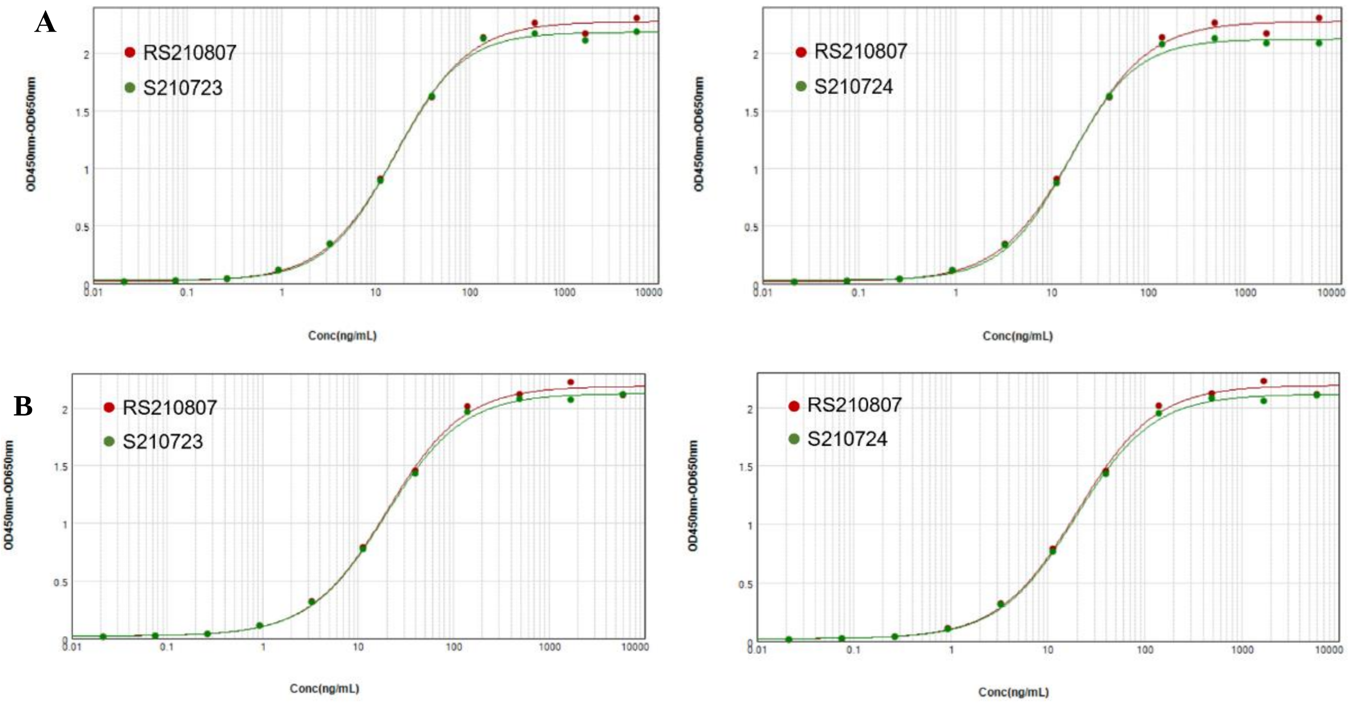

Supplementary Figure 3. Binding testing with antigen A (upper), antigen B (bottom) of antibodies by ELISA. The X axis represented the concentration of test samples. The Y axis represented the signal value detected by OD 450 nm values minus the OD 650 nm values.. RS210807 was settled as reference standard with red curve color, batch S210723 and S210724 were the test pilot samples with green curve color in each figure, respectively.

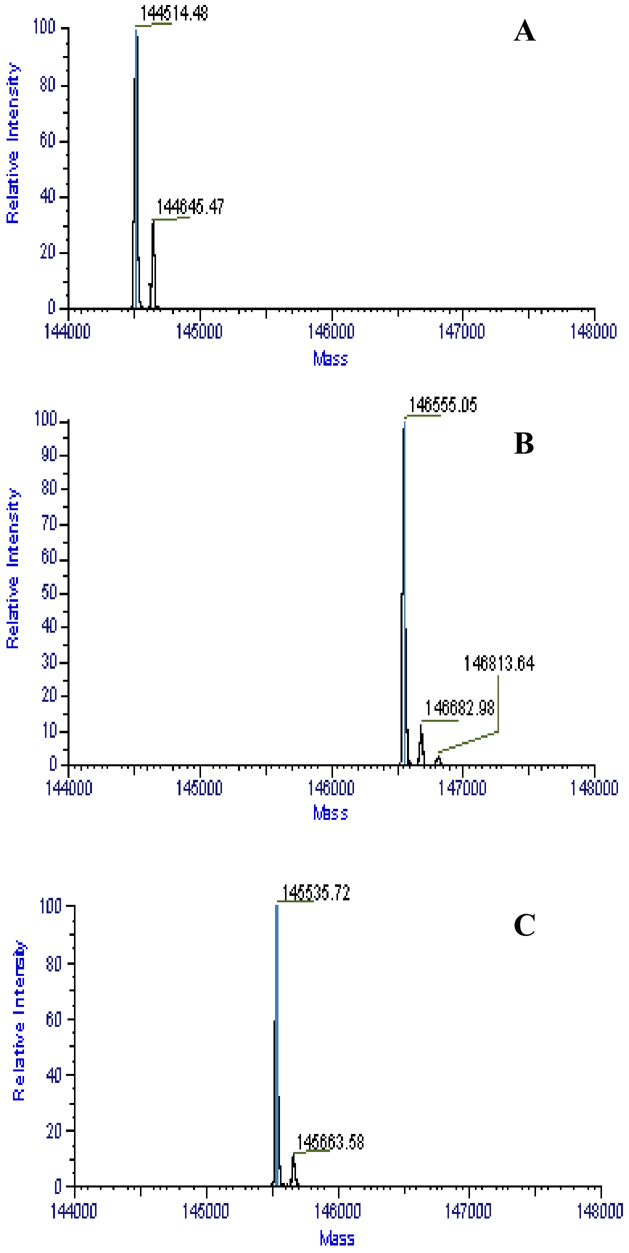

Supplementary Figure 4. Mass spectra for deglycosylated intact mass of (A) parental antibody A, (B) parental antibody B, and (C) bispecific antibody C. The x-axis in each mass spectrum represented the mass-to-charge ratio (m/z) of the ions being analyzed. The y-axis represents the relative abundance of each ion. The detected peaks in Supplementary Figure 4 A, B, C represented the different modified mAbs. The corresponding mass values are listed in Supplementary Table S-6.
